# Glomus Tumor of the Thenar Eminence in Neurofibromatosis Type 1: Case Report and Literature Review

**Published:** 2014-12-19

**Authors:** Gabriele Scaravilli, Roberto Rossi, Stefano Artiaco, Giovanni Merolla

**Affiliations:** 1 Orthopedics and Trauma Clinic, Second University of Naples - Naples - Italy; 2 U.O.C Muscoloskeletal Traumatology, U.O.D. Microsurgery, C.T.O. Hospital, Torino, Italy; 3 Unit of Shoulder and Elbow Surgery, D. Cervesi Hospital, Cattolica - AUSL della Romagna- Italy; 4 Biomechanics Laboratory “Marco Simoncelli”, D. Cervesi Hospital, Cattolica - AUSL della Romagna – Italy

**Keywords:** Glomic Tumor, Neurofibromatosis, Biallelic Inactivation, Finger, Pain

## Abstract

Neurofibromatosis type 1 (NF1) is a disease characterized by increased tumorigenesis susceptibility, caused by mutations of the oncosuppressor gene *NF1*. The glomus tumor (GT) is a rare, very painful mesenchymal neoplasm, arising from the glomus body. In recent years, it has been highlighted the association between NF1 and GT. We report a case of a man aged 65 years, suffering from NF1, with intense pain at the thenar eminence of the right hand, successfully treated with the excision of the mass.

## Introduction

Neurofibromatosis type 1 (NF1) is an autosomal dominant disorder, with an incidence of 1/2,500–3,000 births and a prevalence of approximately 1/4,000–5,000 individuals [1]. It is caused by mutations in the *NF1* tumor suppressor gene, located on chromosome 17 (17q11.2), which encodes neurofibromin (nf), a protein able to downregulate the Ras-Raf/MAPK signaling pathway that activates cell proliferation [2]. Mutations of the NF1 gene result in alteration or loss of function of negative regulator of growth and cellular differentiation of nf, leading to uncontrolled cell proliferation and increased risk of developing cancer [3,4].

The glomic or glomus tumors (GT) are composed of benign mesenchymal neoplasms, arising from glomus cells that are modified smooth muscle cells of the glomus body (Masson glomus), a thermoregulatory structure of the dermis [5].

Glomus bodies are mostly found in the palm of the hand and sole of the foot, reaching the highest concentration in the fingers, especially in the nail bed and the distal phalanx, which are elective sites of glomus tumor [6].

It represents almost 2% of all soft tissues tumors and from 1 to 5% of all hand neoplasms, with high incidence in women aged between 20 and 40 years [7]. The GT generally looks like a solid, encapsulated, spherical mass, located in the deep dermis or subcutaneous tissue, typically measuring 2–3 mm in diameter, but it can reach more than 15 mm. Klaber et al. in 1938 reported the first case of GT in patients with NF1 [8].

The common ultrasonographic (US) features of glomus tumors include a small (≤1.0 cm), solid, homogeneously hypoechoic or isoechoic, well-demarcated nodule; hypervascularity on color or power Doppler imaging; and bony erosion of the underlying phalangeal bone [9].

MR imaging showed localization of a hypointense (dark) lesion that was well marginated and oval-shaped on T1-weighted images. On T2-weighted images, these lesions appeared brighter and hyper-intense with a hypointense rim [10].

In the current study, we report an atypical location of GT of the hand in NF1 and we review the literature.

## Case report

**Figure 1:**
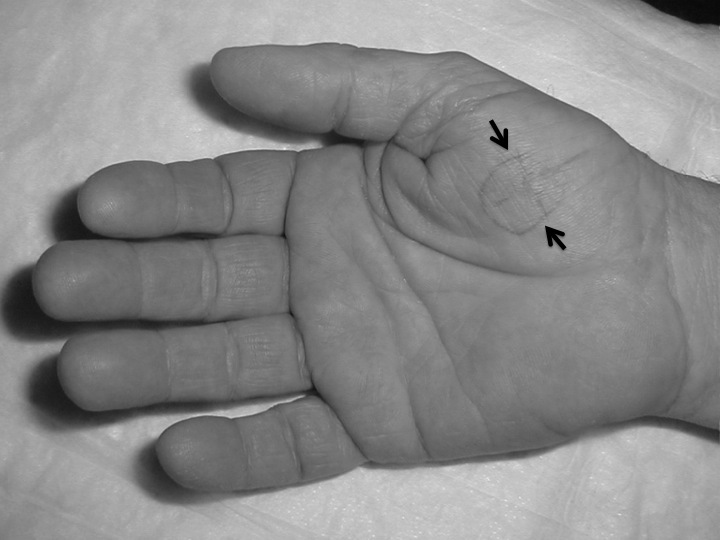
Eminence thenar of the right hand with the areas where the neoformation was found (black arrows) and the Love test was positive.

**Figure 2:**
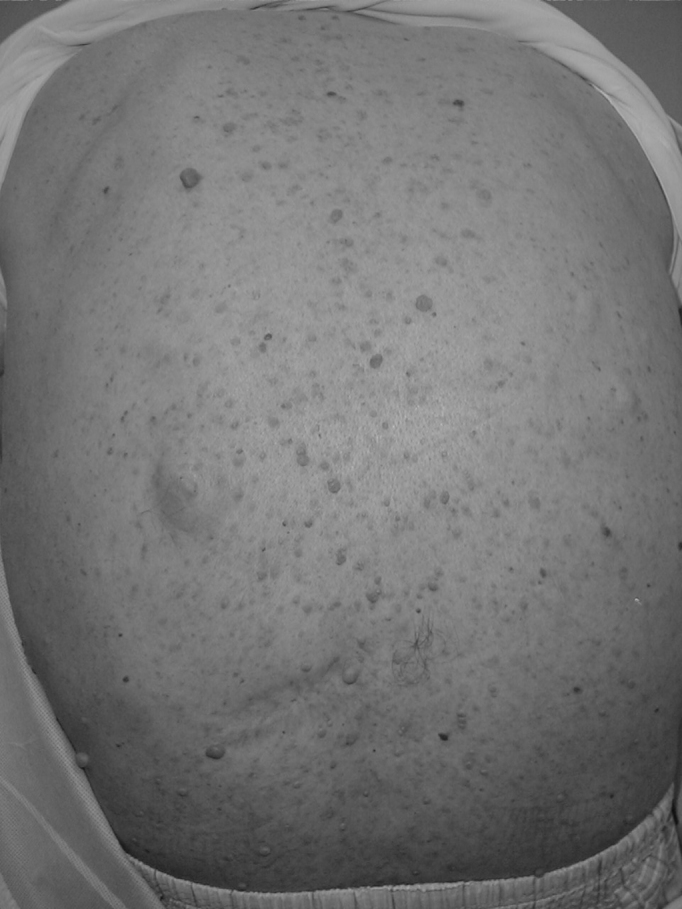
Typical neurofibromas along the trunk and back of the patient

A man aged 65 with NF1 underwent to our institution complaining for severe pain of the thenar eminence in the right hand (Figures 1–2). He had previously consulted other specialists (rheumatologist, physiatrist, neurologist, psychiatrist), without a specific diagnosis.

Pain was triggered by a simple touch and low heat. Inspection highlighted a slight nodular swelling of the thenar eminence without any changes of the color and trophism of the skin (Figure 1).

The signs of triad of Carroll [11] (hypersensitivity to cold [Cold Sensitivity Test], paroxysmal pain, compression pain [Love Test] [12]. Pain was attenuated after application of a tourniquet (Hildreth’s test) [13]. X-ray did not show bony alteration.

**Figure 3:**
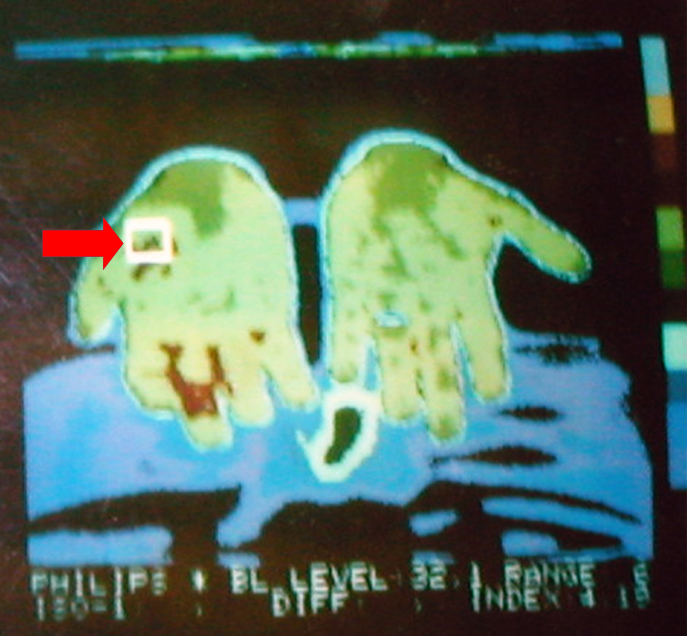
Thermography show the hyperthermic area in the thenar eminence of the right hand where the glomus tumor was located (red arrow).

The vascular origin of the neoformation was confirmed by thermographic examination that recorded an increase of the local heat in an unusual site for GT [14] (Figure 3).

**Figure 4:**
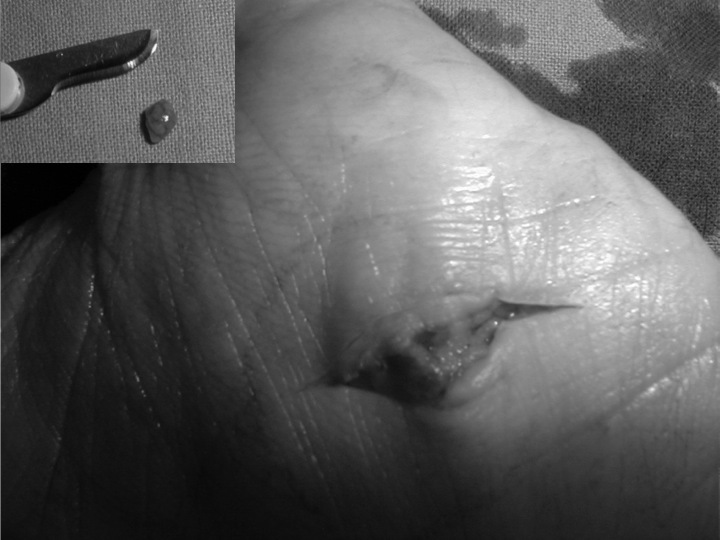
Intraoperative findings. The glomus tumor was excised through a volar approach and was found to to be very shallow. The window on the left top shows the well capsulated neoformation as it appeared at the end of the procedure.

Surgical excision was performed in local anesthesia with an incision of 1 cm at the level of the palpable mass, which was found to be very shallow (Figure 4).

The excised neoformation appeared macroscopically as a solid highly vascularized, encapsulated mass of 3 cm with a reddish-brown coloration (Figure 4). A careful inspection of the surrounding subcutaneous tissues with microsurgical glasses did not show additional anatomical abnormalities.

**Figure 5:**
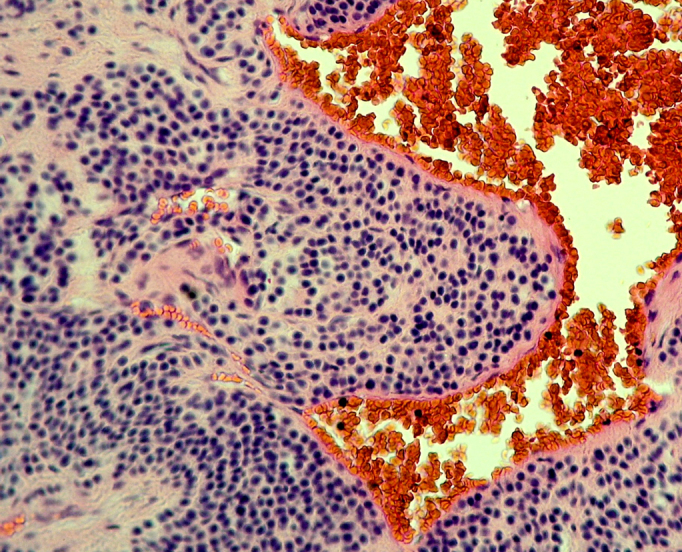
Histological findings: blood vessels surrounded by a proliferation of small round cells with dark nuclei in a myxoid stroma.

The patient had a significant pain reduction within 24 hours and a complete resolution of all symptoms in one week. Histological examination showed blood vessels surrounded by a proliferation of small round cells with dark nuclei in a myxoid stroma, confirming the diagnosis of glomus tumor (Figure 5).

## Discussion

The GT is a rare neoplasm whose incidence is underestimated due to misdiagnosis of the mass as a neuroma, melanoma, gouty tophi, angioleiomyoma, neurofibromas, foreign bodies, ganglions, etc. From clinical history arise a mean duration of symptoms of 10 years and the patient often consult several specialists (orthopedic, rheumatologist, physiatrist, neurologist), including often a psychiatrist, before to achieve the proper diagnosis [15].

Pain and hand dysfunction mainly affect daily manual activities, resulting in a severe deterioration of patient’s quality of life of patients. The intense pain, often referred as “hammer blow”, usually lasts less than a minute, but the quick reappear of the local symptoms generates a state of anxiety pending a new triggering event that may recur.

For these reasons, the patients pose the hand in defense from external stimuli in different ways such as keeping the hand closed in a sleeve, wrapping it in a cloth, or leaving it permanently in a glove. Moreover, the clinical diagnosis is facilitated by the recognition of at least 2 out of 3 symptoms of the aforedescribed triad of Carroll and the positivity of the Hildreth’s Test [16,17].

The Hildreth’s test, easy to perform, has a high sensitivity (71.4%), a specificity of 100% and an accuracy of 78%; the Love’s pin test, although with a sensitivity of 100% and an accuracy of 78%, has a specificity of 0%; the Cold sensitivity Test has sensitivity, specificity and accuracy of 100%. For this reason, it is always advisable to perform all aforementioned tests [18].

Diagnostic imaging, as MRI and US, is of great assistance to a mainly clinical diagnosis, except for those atypical localizations (cutaneous and visceral) [19]. The literature shows a strong correlation between GT and NF1 [20–24].

In patients with NF1, the onset of a GT is related to biallelic inactivation of the NF1 gene, presumably due to errors during DNA replication in mitosis, that leads to the activation of a cascade of growth factors depending on Ras-Raf/MAPK pathway [20]. This signaling pathway stimulates the growth of glomic cells, playing a key role in the development of GT in patients with NF1 [21].

The differential diagnosis is made with neurofibromas, characteristic skin lesion of the NF1, from which it differs in its usual location at the distal phalanges, and the painful symptoms that are triggered by low intensity stimuli.

However, Maertens et al [22] in 2007 showed the presence of a somatic mosaicism in NF1, mainly confined to neural crest cells, or belonging to the neural crest origin, confirmed by molecular analysis. Neurofibromas of Schwann cells and melanocytes taken from the cafe-au-lait spots showed biallelic inactivation of the NF1 gene [23].

Due to the extreme similarity of the pathogenetic mechanisms with neurofibromas and stains, Brems et al [24] hypothesized that the GT might arise from myofibroblasts derived from the neural crest stem cells. Furthermore, it was demonstrated that the silencing of the NF1 gene induces increased expression of vascular endothelial growth factor (VEGF) [25,26], further clarifying the reasons of occurrence of a hypervascularized neoplasm, such as the GT.

In general, NF1-associated glomus tumors share many histologic similarities to sporadic ones: namely the cuboidal cells surrounding blood vessels, smooth muscle actin (SMA) positivity, and benign appearing rounded nuclei with moderate to abundant cytoplasm [27]. Since the presence of GT is underestimated, is not uncommon in the clinical practice to leave these patients with a severe pain for long time.

Individuals with NF1 may be at an increased risk to develop chronic pain syndromes like CRPS since neurofibromin (the protein product of NF1) plays a key role in the excitability regulation of nociceptive sensory neurons [28]. Avoidance or elimination of chronic inciting “pain generators” in CRPS is critical. Specifically in the case of glomus tumors, this is difficult to achieve in practice since the tumors are under-recognized and patients may have symptoms for years or decades before proper diagnosis [29,30].

The recurrence rate following surgery ranged from 0% to 33.3% (30) While early recurrence may occur within weeks to months of surgery and presumably reflects inadequate excision, later recurrence (years) is extremely rare and it is probably the result of the development of a new tumor [31].

Up to date several single report or case series studies on GT have been published [21,24,25,27,32–42].

The first association between GT and NF1 was described on a leg of a young girl by Klaber in 1938 [8]. GT have been restricted in the subungual [32–34], to the fingers [21], fingers and toes [35] or described in multiple and different locations [24,35]. Other researchers described GT in the thumb of a child [36], association between GT and a rare subtype of segmental neurofibromatosis [37] or CPRS secondary to multiple GT of fingertips [38]. Other studies have been recently published (between 2012–2014) [27,25,38,39,40–42] where some authors have also clarified the genetic mechanisms that underlies the association between GT and the subtypes of NF1 [27,25].

The novelty of our report is the atypical localisation in the thenar eminence, compared to other cases described in the literature, that usually affects fingers and toes.

The excision of GT in our patient results in complete pain relief and resolution of clinical symptoms. No recurrence was observed after 2 years from surgery.

No recurrence was observed in 2 years from surgery. We consider that the adoption of microsurgical glasses for inspecting the area after excision, may be helpful to avoid incomplete tumor removal.

GT can be found in about 5% of patients with NF1[32]. In most NF1 clinical programs, a routine screening for GT is not common and individuals with NF1 may experience pain from for up to 20 years before diagnosis [33]. The relative rarity of these tumors, their small size, and their variable presentation often results in misdiagnosis and delayed treatment. The surgical approach reported in this study allow a faster solution, through an early clinical diagnosis, that can avoid the persistent and debilitating pain; furthermore, an early surgical approach can prevent an extensive demolition due to the neoplasm growth, with a minimum patient discomfort, satisfactory cosmetic results and an improved quality of life.
